# Host species heterogeneity in the epidemiology of *Nesopora caninum*

**DOI:** 10.1371/journal.pone.0183900

**Published:** 2017-08-29

**Authors:** Karla I. Moreno-Torres, Laura W. Pomeroy, Mark Moritz, William Saville, Barbara Wolfe, Rebecca Garabed

**Affiliations:** 1 The Ohio State University, Department of Veterinary Preventive Medicine, Columbus, Ohio, United States of America; 2 The Ohio State University, Department of Anthropology, Columbus, Ohio, United States of America; 3 Morris Animal Foundation, Denver, Colorado, United States of America; 4 The Ohio State University, Public Health Preparedness for Infectious Diseases Program, Columbus, Ohio, United States of America; NIH, UNITED STATES

## Abstract

Pathogen transmission across species drives disease emergence; however, mechanisms by which multi-host pathogens cross species boundaries are not well identified. This knowledge gap prevents integrated and targeted control in an epidemiologically continuous ecosystem. Our goal is to describe the impact of host species heterogeneity on the epidemiology of *Neospora caninum* circulating between livestock and wildlife in southeastern Ohio. We collected biological samples from Père David’s deer (*Elaphurus davidianus*) located at an outdoor wildlife conservation center; from cattle raised at farms adjacent to the center; and from wild white-tailed deer that roamed across farm and center boundaries. We designed nested infectious disease models of competing hypotheses about transmission and used collected data to fit the models, thereby estimating important immunological and transmission quantities which describe the species-specific contribution to the persistence of this pathogen in the community. We applied these data and models to suggest appropriate species-specific disease control methods. Results show that immunity in cattle and Pére David’s deer wanes over time, while in white-tailed deer immunity appears to be lifelong. Transmission quantities for cattle were estimated at values below the threshold for an outbreak (R_*t*_ < 1), meaning that chains of transmission are not maintained within this population and infections must occur due to reintroduction from an outside source. Pére David’s deer and white-tailed deer both could maintain continuous chains of transmission within their group (R_*t*_ > 1). Therefore, we propose that control of contact with outside sources will be useful for disease control in cattle; boosting immunity with vaccines might be an avenue to prevent infection in cattle and Père David’s deer. White-tailed deer are a potential maintenance host for infection and require further study to determine optimal control methods. Community-level investigations like this allow us to better evaluate heterogeneities in transmission processes that ultimately guide targeted control.

## Introduction

Most pathogens that threaten human and livestock populations persist by infecting multiple species [1]. These are called multi-host pathogens. The enormous impact of multi-host pathogens has been quantified: >61% of human pathogens, >77% of livestock pathogens, and >90% of carnivore pathogens participate in cross-species transmission events [1, 2]. Nevertheless, most research on multi-host pathogens investigates only one part of the system by considering either unidirectional transmission or single-host pathogens [1, 3]. This gap in research may result, in part, from the difficulty in identifying the role of each host species in multi-species disease transmission dynamics. Each host species can be identified as a maintenance population, as a part of a multi-species reservoir population, or as both. Maintenance populations are defined as closed populations that can maintain chains of transmission because they exceed the critical community size [4, 5]; reservoirs are epidemiologically connected populations or environments–including both maintenance and non-maintenance populations–in which pathogens can be maintained and transmitted to populations of interest [4]. Interventions to control and prevent disease, logically, will differ depending on how the reservoir is constituted and which species act as maintenance populations. Thus, understanding host species heterogeneity in the transmission dynamics of multi-host pathogens is essential for targeted control [6–8].

In natural settings, quantifying host species heterogeneity is often confounded by multiple sources of interspecies variation, including immunity, management, behaviors, and age structure of herds or packs. One solution for determining host species heterogeneity–even with species-specific confounders–is to estimate key transmission parameters using infectious disease models statistically fit to serology data collected from multiple species. A key parameter is the *force of infection* (FoI), defined as the per-capita rate at which susceptible individuals acquire infection, which can quantify heterogeneities in transmission [9]. In addition, the FoI can be used to calculate the basic reproductive number (*R*_*0*_), which is defined as the average number of secondary cases arising from an average primary case in an entirely susceptible population. *R*_*0*_ is a critical metric that can inform the maximum transmission potential for an infectious disease in a population. Thus by estimating *R*_*0*_ for each population that constitutes the community, the species-specific transmission of a pathogen in that community can be compared, and targets for disease control can be established [9, 10].

We use the case study of *Neospora caninum*, a protozoan parasite capable of infecting a wide range of canid and ungulate species [11–13] to quantify host species heterogeneity of this understudied, but financially important, multi-host parasite [14–16]. The disease is important due to the substantial economic losses the parasite causes to the national and international cattle industry, mainly derived from premature culling, reduced milk yields and abortions [17, 18]. *N*. *caninum* is estimated to cost $843 million dollars annually in US dairy farms alone [17].

The life cycle of *N*. *caninum* involves three known stages. Oocysts are shed to the environment in feces by the definitive host–i.e., dogs and wild canids–and consumed by the intermediate host, which are primarily ruminants such as cattle and deer. Tachyzoites and bradyzoites develop in the intermediate host, causing damage to infected tissues and resulting in abortion, maternal infertility, and other clinical signs [13]. Prevention of *N*. *caninum* infection in cattle is based on reducing direct and indirect interactions between the definitive host (canids) and the intermediate host (ruminants) [19]. Also, because transplacental transmission from cow to calf seems to be the primary source of maintaining the parasite within the herd as opposed to horizontal transmission via colostrum and/or interactions with canids, maintaining a closed herd [19] and vaccination [20] have been suggested as control strategies to limit infection.

In current practice, however, there is no vaccine nor effective treatment on the market for *N*. *caninum* in cattle [21]. To our knowledge, current control and prevention strategies are not based on actual studies of transmission across species. Furthermore, neither identification nor inclusion of other plausible hosts, such as rodents and birds, have been explored as part of a control strategy that would target an *N*. *caninum* reservoir. Disease control currently targeting cattle populations may be targeting part of the reservoir community, but might not be targeting maintenance populations. To move forward with disease control, identification of maintenance populations is crucial.

In our study area in southeastern Ohio, differences in estimated true prevalence for intermediate ruminant host species suggested differences in the epidemiology of *N*. *caninum* for these interacting populations [22]. To understand why those differences were found, an evaluation of the environmental phase of *N*. *caninum* shed in wild canid scats was pursued. Results suggested that in our study population, environmental transmission to ruminants is likely rare [23]. Consequently, our next logical step was to evaluate host species heterogeneity in the epidemiology of *N*. *caninum* in ungulates in the community.

Therefore, our goal is to better understand the role of host species heterogeneity in the epidemiology of *N*. *caninum* circulating in a community. We evaluated transmission dynamics and immunodynamics in three intermediate ruminant host species by: 1) estimating and comparing their FoI; and 2) estimating the duration of immunity. Also, we estimated the species-specific transmission by calculating the reproductive number of each of the three ruminant host species. Results of this study can identify maintenance populations and inform control strategies at the community level.

## Materials and methods

### Study area and population

The ecological system is located in southeastern Ohio and contains our focal species: cattle (*Bos Taurus*), Père David’s deer (*Elaphurus davidianus*) and white-tailed deer (*Odocoileus virginianus*). This region is rural and includes the largest wildlife conservation center in North America, the International Center for the Preservation of Wild Animals (DBA, *the Wilds*). Several rare and endangered ruminant species are bred at the *Wilds* as part of their conservation program. Beef cattle farms predominate in the area surrounding the conservation center, but dairy cattle and small ruminant farms also are found in the community. In addition, farms keep domestic dogs primarily as companions or livestock guards. White-tailed deer in this community are free-ranging and capable of crossing fences and comingling with both domestic and non-domestic captive herds. Free-ranging wild carnivores such as coyote and fox in the area are considered pests, tourist attractions, and a recreation source for the local community. This ecosystem allows us to explore mechanisms of the persistence of multi-host pathogens, such as *N*. *caninum*, across the wildlife-livestock interface in a community of adjacent and intermingling populations.

### Sample collection

In a cross-sectional study design, we collected jugular vein blood samples (10 ml per individual) during March 2013 for 37 (23 females, 13 males, 1 unrecorded) Père David’s deer managed at *the Wilds*. Birth date records were accessed from *the Wilds* database; thus, individuals’ age was calculated from the birth date to the bleeding date. For 137 female cattle, tail vein blood samples (10 ml per individual) were collected at the Muskingum Livestock Auction, from June 2014 to June 2015 (see below for cattle selection methods). In addition, we recorded breed, reproductive status, fetus gestational age and cow age. Reproductive status and gestational-fetal age [24] were estimated via rectal palpation by the auction veterinarian, and cattle age was determined through dentition [25] by an experienced worker at the auction. The same veterinarian and worker evaluated all cattle during the entire study. Forty-two free-ranging white-tailed deer (37 females and 5 males), with approximately 4 (depending on gender and the number of fetuses) samples collected from each animal (blood, brain, placenta and fetal tissues) were obtained from the study area by volunteer hunters during Ohio’s hunting season (October–February) in 2012, 2013 and 2014. We collected 10ml of blood per deer directly from the heart soon after death, to avoid degradation of antibodies. We estimated individuals’ ages through dentition [26], recorded reproductive status based on visual inspection of the uterus, ovaries, and estimated gestational-fetal age through crown-rump length measurements [27].

Cattle selection at the auction was conducted systematically, stratified based on township, and weighted based on approximate numbers of cattle farms in each township using estimates provided by the county extension agents in the area (Muskingum, Morgan, Guernsey and Noble counties, OH; Table B in S1 Text). We continued to sample cattle arriving at the auction from the targeted townships until the sample size goal for that township was achieved. Additionally, we limited our sampling to a maximum of three cows selected per owner/seller per auction day. Targeted townships were located within 25 km radius from *the Wild*s. The area selected in this study (25 km radius) was based on the average home range of coyotes (17.5 km^2^) and follows previous study design [23]. The auction was used for sampling because adequate management facilities do not exist on most farms in this area to effectively restrain cattle for blood collection. The cattle at auction are not a random sample of all cattle in the area–cattle at auction tend to be young healthy beef cattle and culled breeding/ dairy stock. Therefore, we limited our population to adult cows.

### Laboratory testing

Serum was separated by centrifugation and collected tissues were frozen. Serum and tissues were stored at -20°C. All species’ serum was tested for *N*. *caninum* antibodies using a commercial cELISA kit (VMRD, Inc., Pullman, WA, USA) and white-tailed deer tissues were tested for *N*. *caninum* DNA by using species-specific primers: Np4-Np7 targeting the *pNC-5* gene of *N*. *caninum* through PCR [28, 29] at the Ohio Department of Agriculture Animal Disease Diagnostic Laboratory, Reynoldsburg, OH. The cELISA was performed according to manufacturer recommendations. Individual results were reported as percentage inhibition values; a value ≥ 30% inhibition was considered a positive result and <30% a negative result, as that cut-off is currently used by the manufacturer and has been validated for cattle [30] and for Père David’s deer and white-tailed deer [22]. For each white-tailed deer, three tissue samples were tested (each 20 mg): 1) pooled brain (frontal lobe, cerebellum, cerebral cortex, and spinal cord), 2) pooled placenta, cotyledon and uterus, (multiple areas of each organ), and 3) pooled fetal tissue (brain, lung, heart, kidney, and liver).

### Descriptive data analysis

Descriptive statistics were calculated regarding breed, reproductive status, and gestational-fetal age for cattle, and reproductive status and gestational-fetal age for white-tailed deer. Data on reproductive status was not available for Père David’s deer. Age distribution of each species was also described. In addition, a logistic regression analysis was performed in R [31] to estimate associations between sero-positivity and the potential confounders described above.

### Transmission model implementation, selection, and parameterization

For each species sampled, we considered eight models for *N*. *caninum* transmission: the catalytic model; the catalytic model with maternal antibodies; the catalytic model with age-specific FoI; the catalytic model with maternal antibodies and age-specific FoI; the reverse catalytic model; the reverse catalytic model with maternal antibodies; the reverse catalytic model with age-specific FoI; and the reverse catalytic model with maternal antibodies and age-specific FoI [32, 33]. For cattle, models with maternal antibodies were eliminated because sampled individuals were at least two years of age, and therefore beyond the lifespan of maternal antibodies.

The catalytic model categorizes animals into two states–seronegative or seropositive–assuming that individuals are born into the seronegative state, convert to the seropositive state at the rate designated by the FoI, and remain in the seropositive state due to the lifelong presence of a protective immunological response. Mathematically, let λ represent the constant FoI and let *ɑ* represent the age of each individual in years. According to the catalytic model [32, 33], the age-specific seroprevalence is given by

$$P(ɑ)=1-e^{-\int_{0}^{ɑ}\lambda dɑ}.$$ (1)

Let *t* represent the age at which maternal antibodies wane. Then, the age-specific seroprevelenace can be calculated by the catalytic model with maternal antibodies, such that

$$P(ɑ)=1-e^{-\int_{t}^{ɑ}\lambda dɑ}$$ (2)

In the catalytic models with age-specific FoI, we followed Eqs (1) and (2) with the only difference being that instead of a constant FoI, we fit the data using a b-splined FoI with two equally spaced internal knots.

The reverse catalytic model, similarly, categorizes individuals as seronegative or seropositive, but relaxes the lifelong immunity assumption and allows us to evaluate waning immunity [33]. We assume that immunity lasts for an average duration of 1/ω and then wanes [32]. Again, let λ represent the constant FoI and let *ɑ* represent the age of each individual in years. Then, the age-specific seroprevalence is given by

$$P(ɑ)=\frac{\lambda }{\lambda +\omega }(1-e^{-\int_{0}^{ɑ}\;(\lambda +\omega )dɑ}).$$ (3)

Let *t* represent the age at which maternal antibodies wane. Then, the age-specific seroprevalenace can be calculated by the reverse catalytic model with maternal antibodies, such that

$$P(ɑ)=\frac{\lambda }{\lambda +\omega }(1-e^{-\int_{t}^{ɑ}(\lambda +\omega )dɑ}).$$ (4)

In the reverse catalytic models with age-specific FoI, we followed Eqs (3) and (4) with the only difference being that instead of a constant FoI fit to the data, we used a b-splined FoI with two equally spaced internal knots.

We estimated the FoI and the rate of waning immunity by maximizing the binomial likelihood of seropositive animals, such that

$$y_{a}=Bin(N_{a},p_{a}),$$ (5)

where *N*_*a*_ is the number of animals sampled at each age, *p*_*a*_ is the number of seropositive animals at each age, given in Table 1. We fit candidate models to serology data independently for each species following the method of Pomeroy *et al*. [33]. It is reasonable to assume independence because *N*. *caninum* is not transmitted directly (horizontally) between ruminant species. Likelihood estimations were performed using the “optim” function in R [34]. To select the model that best fit the data for each species, we used the Akaike’s Information Criterion (AIC) [35]. Therefore, we can assess which immunological and transmission processes best described each species.

**Table 1:** Number of samples positive using apparent and true prevalence, by species and age.

*Species*	*Age (years)*	*Apparent Prevalence (AP)*	*True Prevalence (TP)*	*Total sampled*
*Cattle*	**2**	0	1	6
	**3**	1	1	7
	**4**	2	1	6
	**5**	1	1	16
	**6**	1	1	13
	**7**	1	1	14
	**8**	2	2	18
	**9**	1	1	10
	**10**	1	1	16
	**11**	1	2	23
	**12**	1	1	8
*White-tailed deer*	**0.5**	2	2	3
	**1.5**	3	2	4
	**2.5**	3	4	10
	**3.5**	6	7	14
	**4.5**	0	1	3
	**5.5**	3	3	5
	**6.5**	2	2	3
*Père David’s deer*	**1**	2	1	2
	**2**	4	2	6
	**3**	1	1	4
	**4**	5	2	6
	**5**	3	1	4
	**6**	2	1	4
	**7**	0	0	1
	**8**	4	1	4
	**9**	1	0	1
	**10**	0	0	0
	**11**	0	0	0
	**12**	0	0	0
	**13**	0	0	0
	**14**	1	1	2
	**15**	2	1	2
	**16**	0	0	1

To estimate true prevalence, informative prior distributions from models a7, b4 and c4 were used from [22]. The prior prevalence for model a7 was modified for prevalence of cattle in Ohio [36], Beta (alfa = 7.039, beta = 69.479). The mode of the overall cattle population TP was 7.2% (95 PI% 3.9–12%), for Père David’s deer population TP was 53% (95 PI% 39.4–66%) and for white-tailed deer population TP was 53.5% (95 PI% 36–78%). The overall cattle population AP was 8.8% (95 PI% 5.1–14.7%), for Père David’s deer population AP was 67.6% (95 PI% 51.5–80.4%) and for white-tailed deer population AP was 45.2% (95 PI% 31.2–60.1%).

Positive test results can be used to fit models either as apparent prevalence (AP) or as true prevalence (TP). Commonly, AP is used in investigations, however biased conclusions on epidemiological parameters are plausible [37, 38]. Therefore, to compare the impact of fitting models with AP or TP on epidemiological parameters, first, we fit the AP, which is the proportion of animals that tested seropositive at each age. Second, we fit the TP, which is the true proportion of animals seropositive at each age when accounting for diagnostic error. Thus, each model was run for both AP and TP data. We calculated age-specific AP by using seropositive test results by age as the numerator and the total of animals sampled by age as the denominator. We calculated age-specific TP by using Bayesian inference methods with informative prior distributions explicitly described in Moreno-Torres et al. (2016) [22].

We calculated the reproductive number (R_t_), the number of secondary cases produced by a primary infectious case, by multiplying the estimated FoI and the average life expectancy of each species [39]. Finally, we estimated the critical proportion that needs to be vaccinated to prevent an outbreak as a P*c* = 1-(1/R*t*), assuming that the vaccine is completely protective [39]. As most vaccines are not completely protective, P*c* can be thought of as the proportion of the population that must be immunologically protected to prevent an outbreak, and the proportion to be vaccinated can be adjusted based on vaccine efficacy.

### Ethics statement

Animal use protocols were reviewed and approved by the Ohio State University Institutional Animal Care and Use Committee (Protocol Number: 2012A00000154). Both captive species (cattle and Père David’s deer) were physically restrained in a chute and, once restrained, the procedure lasted about 10–15 minutes per animal. Individuals were sampled early in the morning to avoid heat stress and acutely stressed individuals were removed from the study (one cow). A white-tailed deer scientific collection permit was granted by the Division of Wildlife, Ohio Department of Natural Resources (Permit Number: 14–266). The collection of white-tailed deer samples was opportunistic during Ohio’s hunting season; thus, the accessibility and availability of sample collection was restricted to the number of hunters that verbally consented to provide samples and their bag limits. *The Wilds* and the Muskingum Livestock Auction gave written consent to participate in this study. Both facilities provided specialized personnel to handle animals and the necessary equipment to physically restrain animals. Cattle owners/sellers verbally consent cattle sampling at the auction and provided the township location where the cattle resided prior to the auction.

## Results

**Fig 1:**
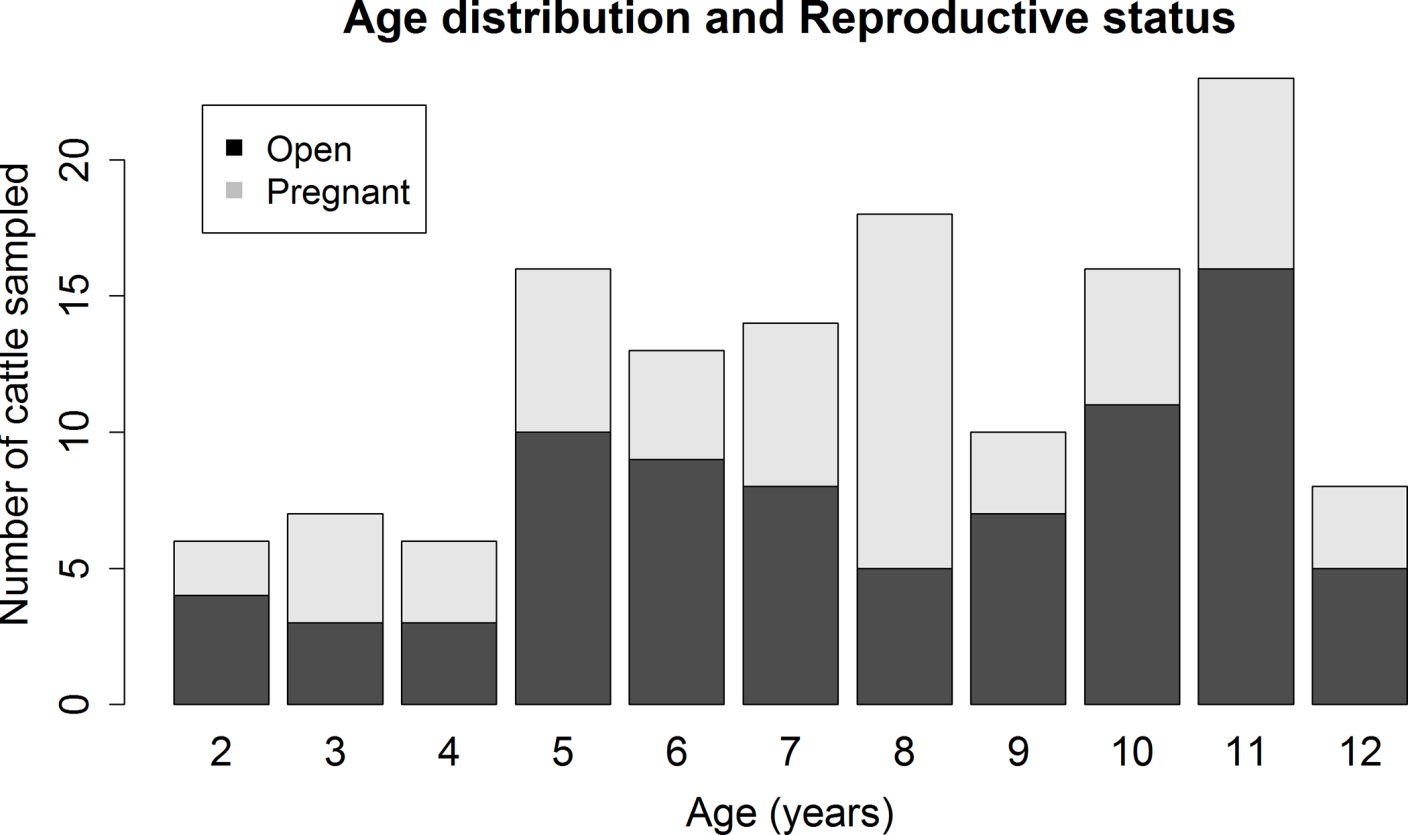
Age distribution and reproductive status of the 137 sampled female cattle. A total of 59% of cattle were open and 41% pregnant.

**Fig 2:**
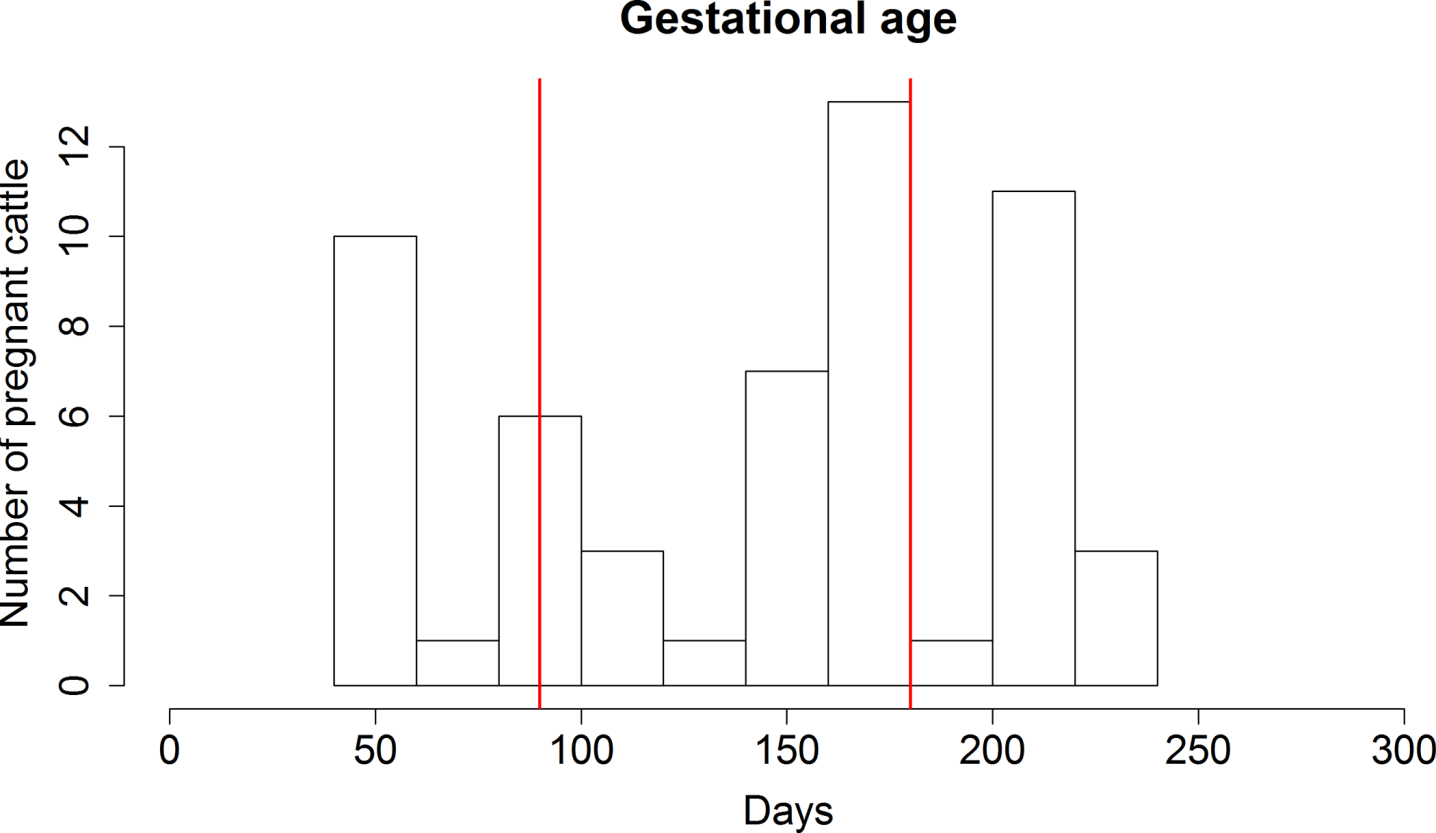
Gestational age of pregnant cattle. Pregnant cattle were distributed in all three trimesters of gestation, indicated by the vertical red lines.

**Fig 3:**
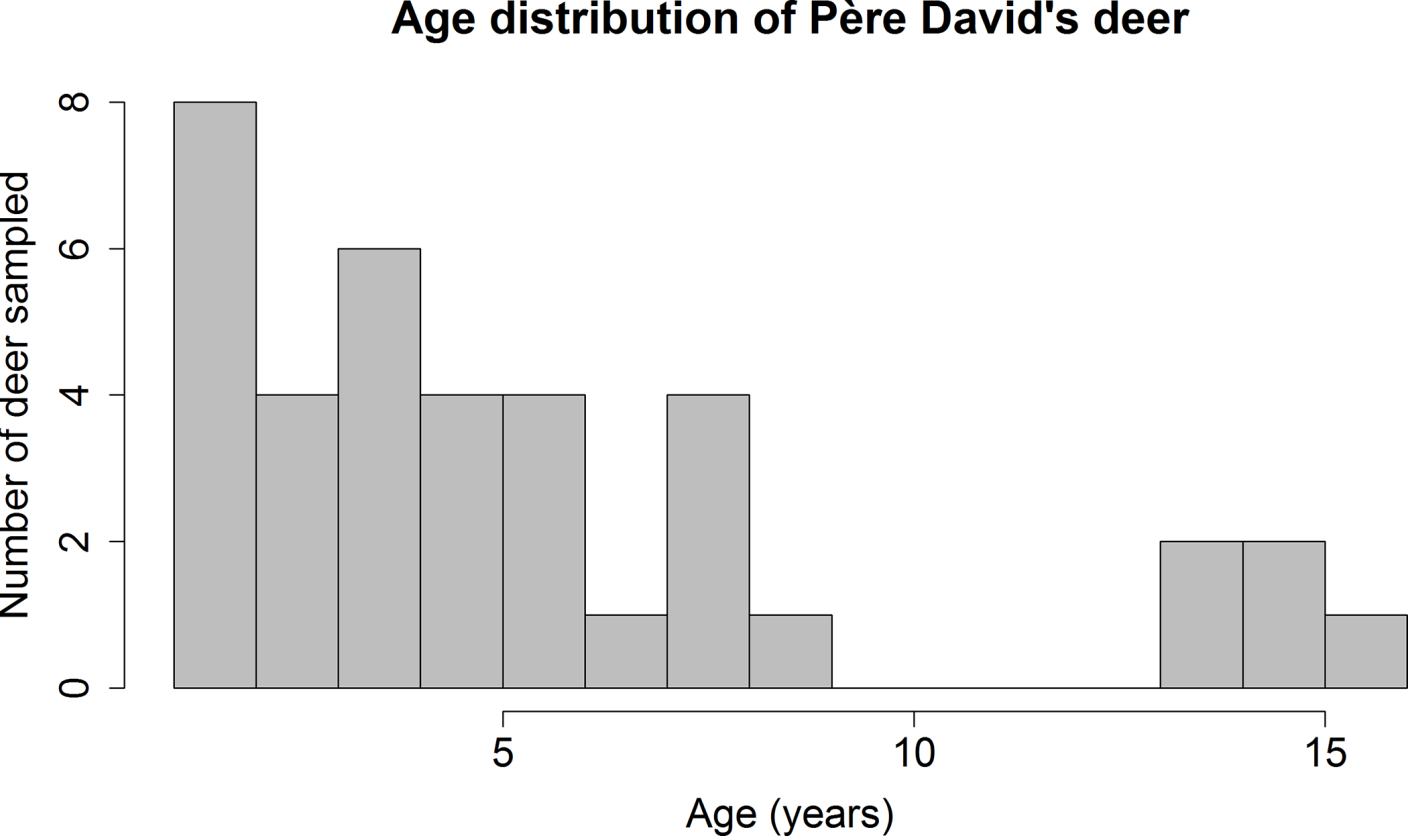
Age distribution of the 37 (23 females, 13 males, 1 unrecorded) sampled Père David’s deer. There were no individuals from 9 to13 year old. Reproductive status was not recorded but pregnant individuals were in their third trimester of gestation, by The *Wilds* records and based on seasonality of breeding in this species.

**Fig 4:**
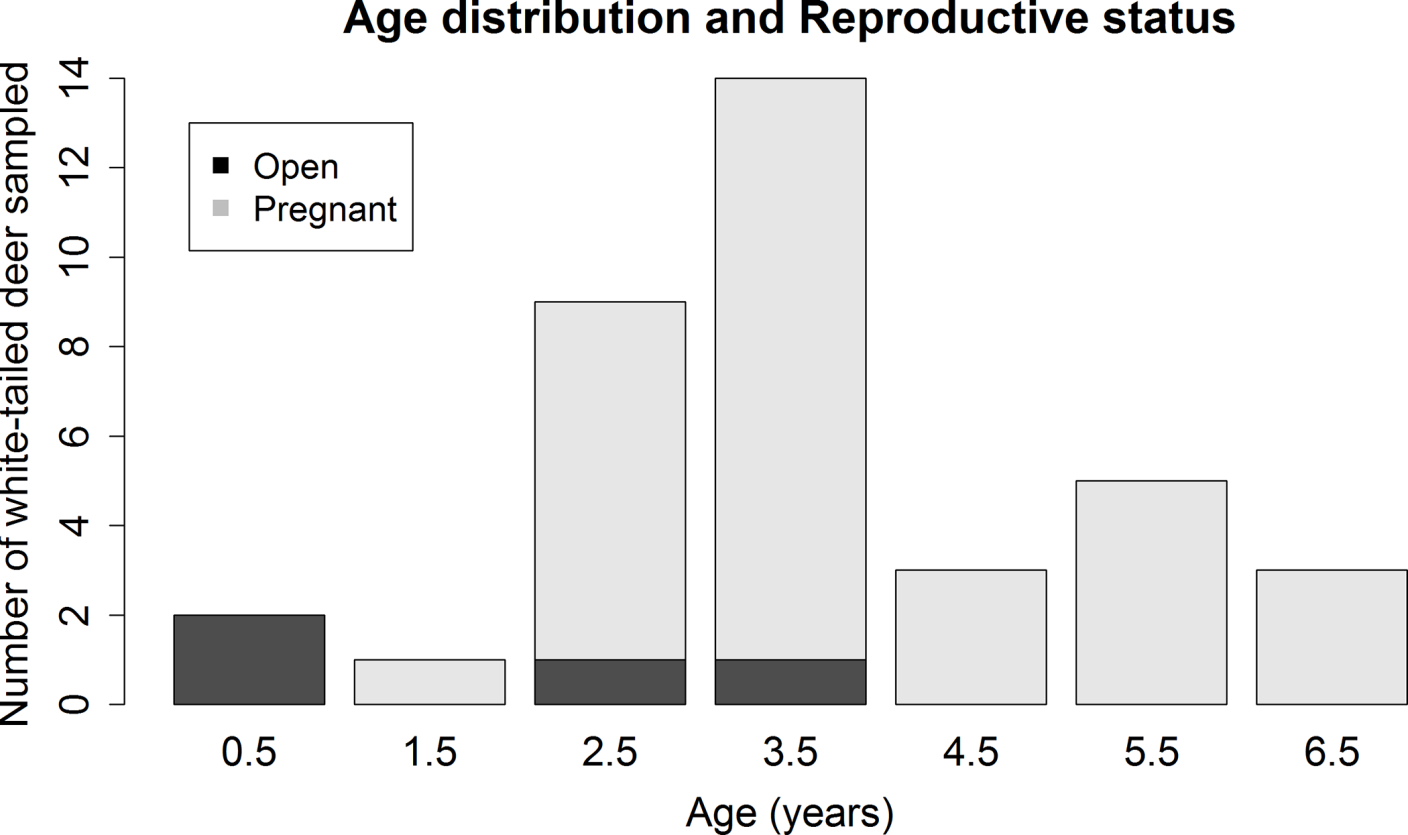
Age distribution and reproductive status of the 37 sampled female white-tailed deer. There were 5 males sampled: 0.5 year old (n = 1), 1.5 year old (n = 3) and 2.5 year old (n = 1) (not displayed in this figure). A total of 11% of female white-tailed deer were open and 89% pregnant.

**Fig 5:**
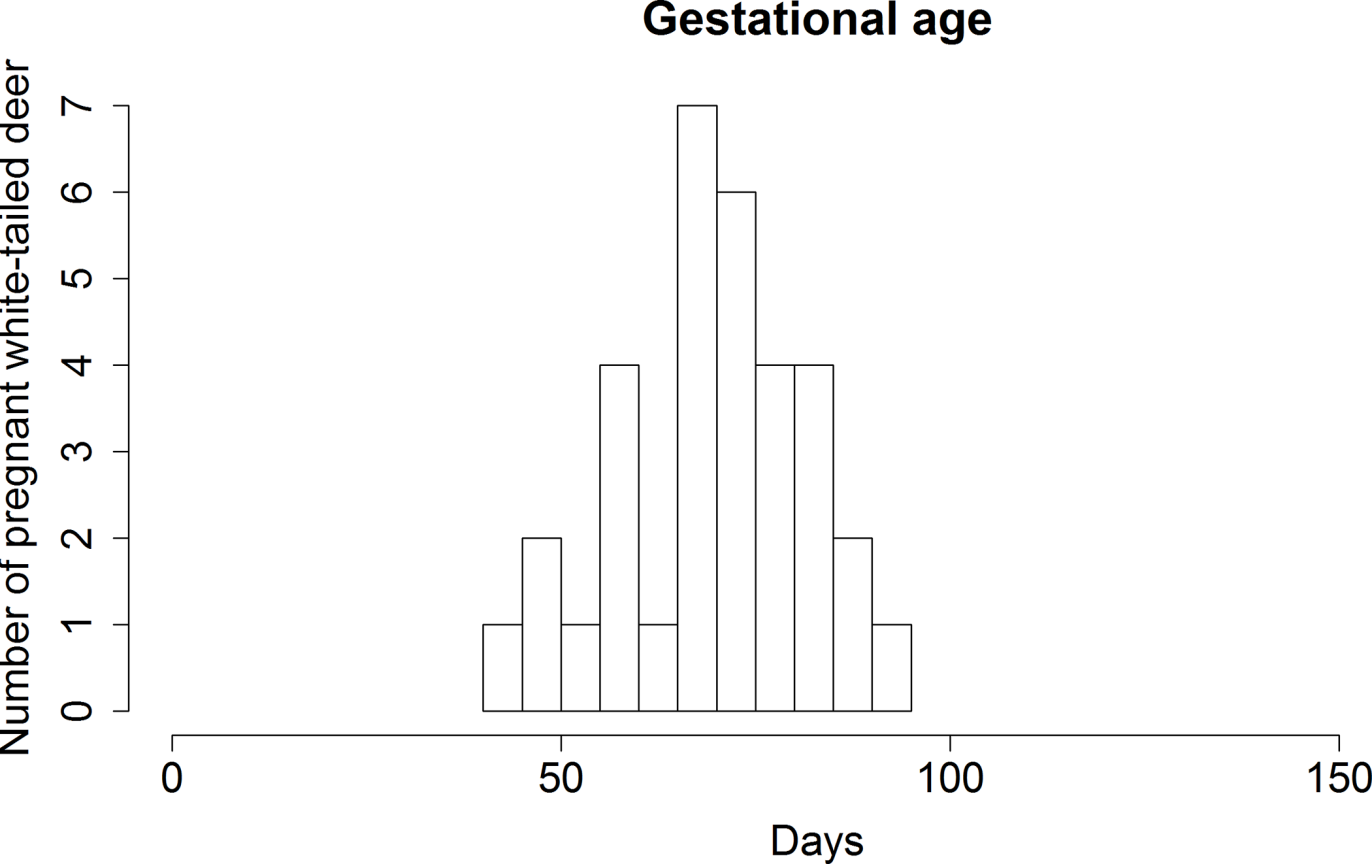
Gestational age of pregnant white-tailed deer. Pregnant white-tailed deer were all in the second trimesters of gestation.

Descriptively, the three sampled ruminant populations varied in age distribution, reproductive status, and gestational age (Figs 1–5). For the 137 cattle sampled, 10% were dairy breeds, 25% crossbreed, and 65% beef breeds (Table C in S1 Text). In logistic regression models, there was no association between seropositivity and the covariates breed, reproductive status and breed, or gestational age and breed for cattle or white-tailed deer. As reproductive data were not available for the Père David’s deer, similar associations could not be tested in this species.

**Fig 6:**
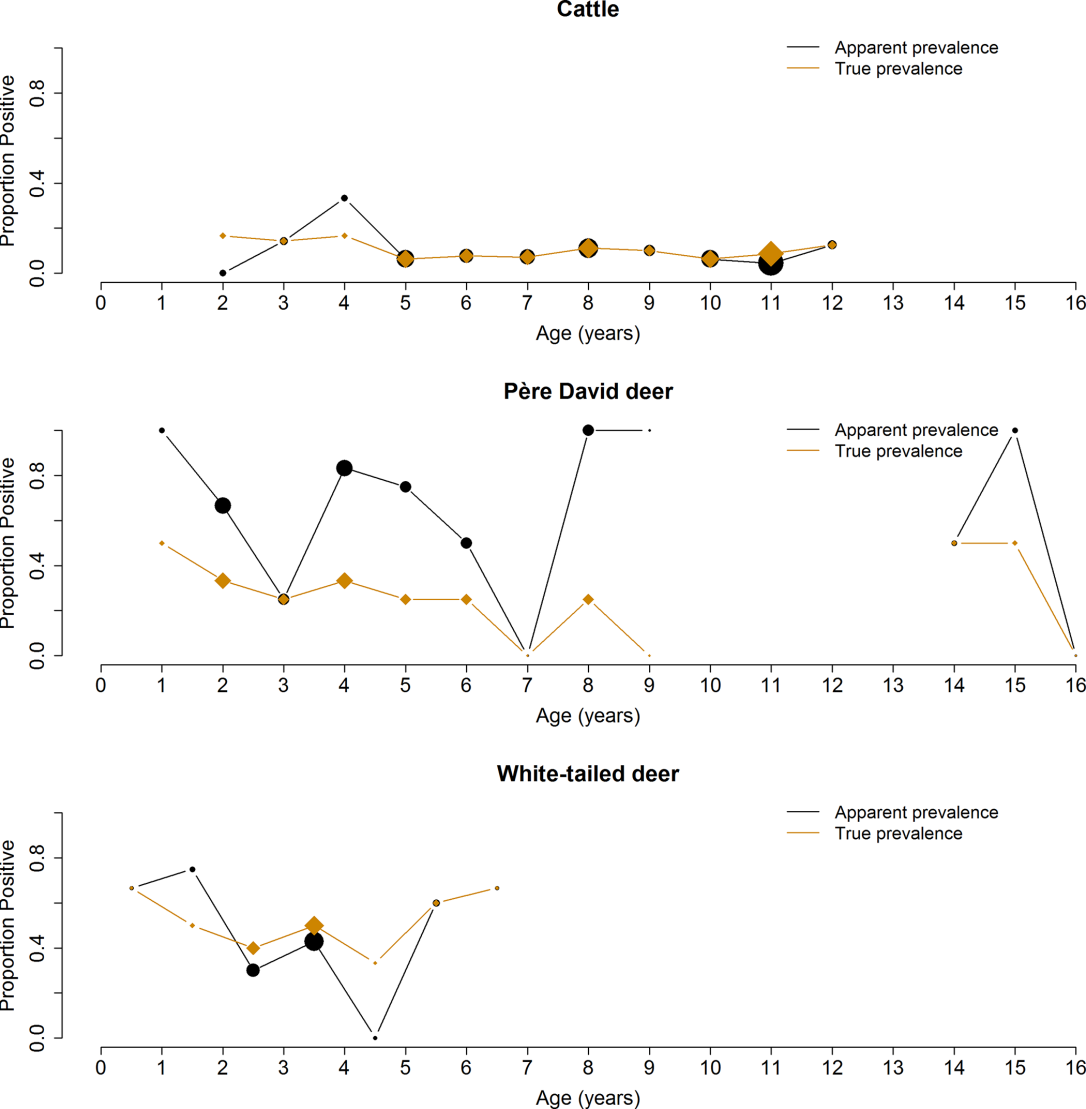
Apparent and true prevalence of proportion positive individuals by species and age. The size of the circles or diamonds is proportional to the number of animals sampled at each age.

To determine the prevalence of *N*. *caninum* in white-tailed deer, both jugular vein blood samples and tissue samples were collected from free-ranging animals. These samples were analyzed using PCR and cELISA. PCR tests for the presence of DNA, giving information about active infection, latent or recently degraded DNA; however, PCR is more likely to give false negative results if a low load of parasites is present and if tissue selection is arbitrary rather than focused in histopathology results. The cELISA tests for the presence of antibodies–with inhibition above a predetermined threshold considered seropositive–indicating if a past exposure occurred but not necessarily providing information on infection. All white-tailed deer tissue samples were negative for *N*. *caninum* DNA by PCR test. These negative PCR test results contrast with the cELISA test results, of which 45% tested positive (overall AP). The proportion of the white-tailed deer population that tested seropositive by cELISA varied by age (Fig 6). These results suggest that the white-tailed deer sampled were infected with the parasite sometime in the past but were not experiencing active or latent infection at the time of sampling.

To determine the prevalence of exposure of *N*. *caninum* in cattle and Père David’s deer, cELISA tests were performed. Like the white-tailed deer, both cattle and Père David’s deer showed evidence of seropositivity, 8.7% overall AP and 67.5% overall AP, respectively. The proportion of seropositive individuals per age by species is shown in Fig 6. These results suggest that both cattle and Père David’s deer were infected with the parasite sometime in the past.

The estimated TP of *N*. *caninum* in all three species differed from AP. These differences between AP and TP were expected, given that we are accounting for the diagnostic error in the TP. The mode of the overall cattle population TP was 7.2% (95 PI% 3.9–12%), for Père David’s deer population TP was 53% (95 PI% 39.4–66%) and for white-tailed deer population TP was 53.5% (95 PI% 36–78%).

**Fig 7:**
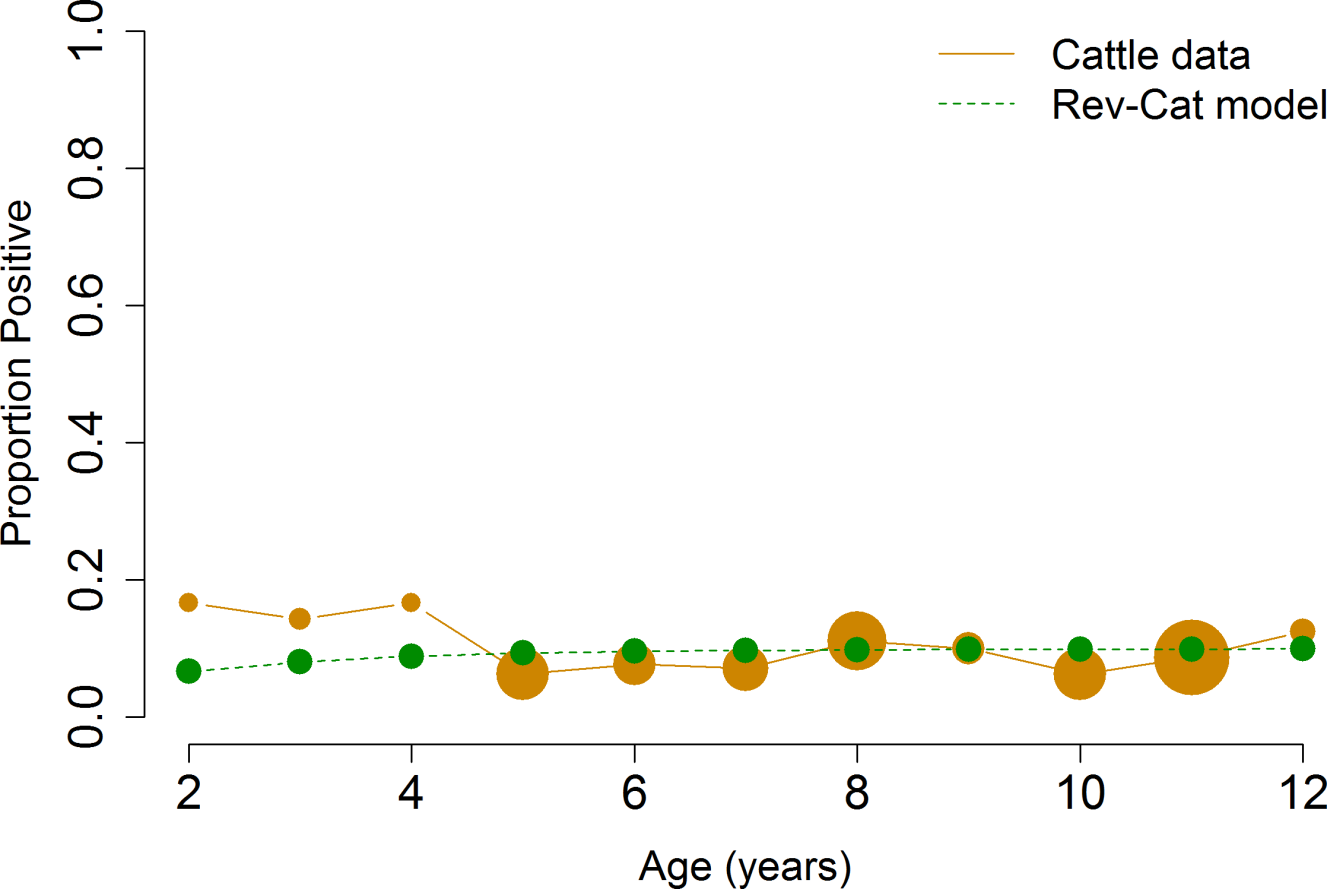
Model selection for cattle. Reverse catalytic model was the best-fit model for cattle. Size of points represents the relative number of animals sampled with that age.

**Fig 8:**
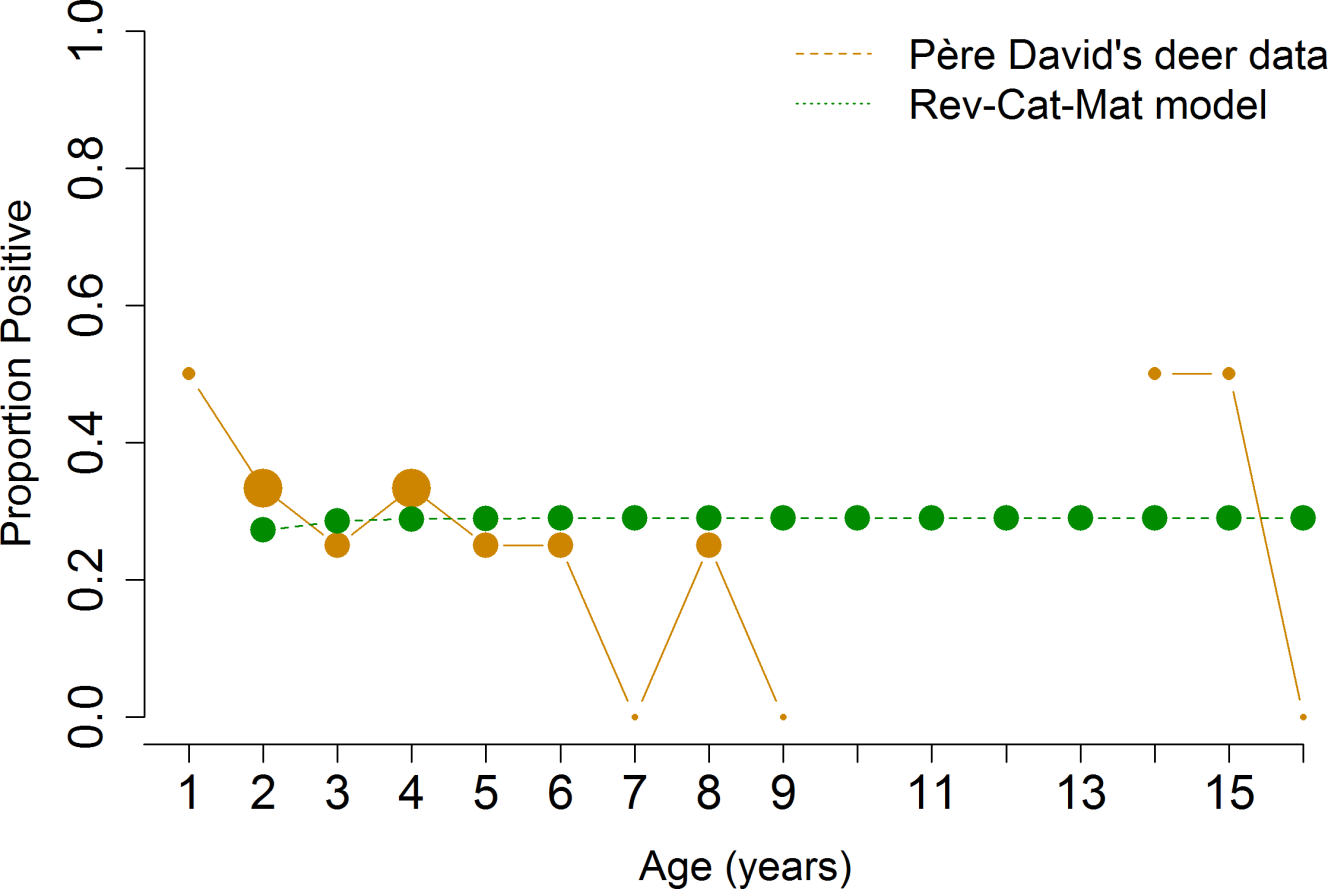
Model selection for Père David’s deer. Reverse catalytic model with maternal antibodies was the best-fit model for Père David’s deer. Size of points represents the relative number of animals sampled with that age.

**Fig 9:**
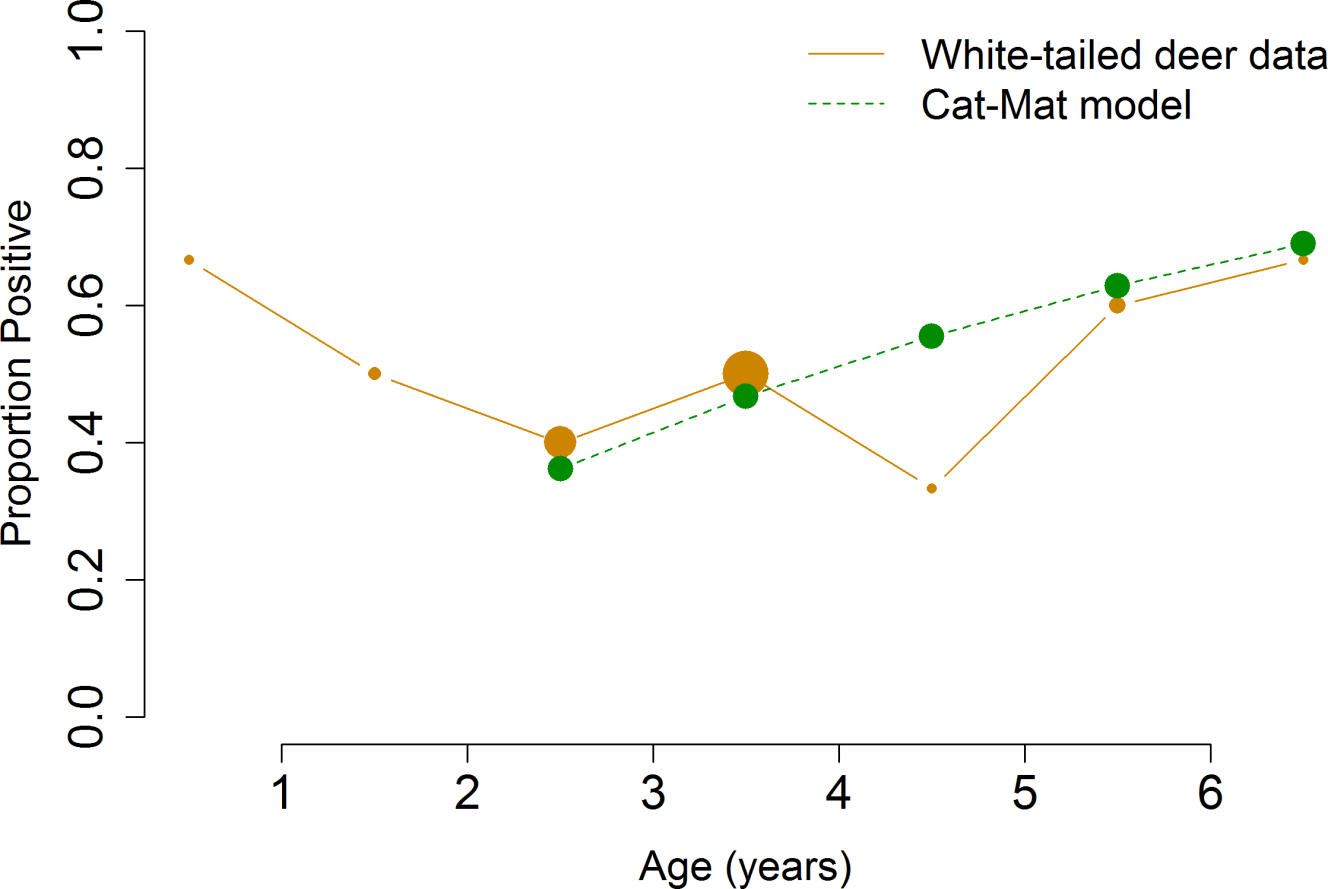
Model selection for white-tailed deer. Catalytic model with maternal antibodies was the best-fit model for white-tailed deer. Size of points represents the relative number of animals sampled with that age.

To quantify the duration of seropositivity for each host species, we fit age-structured serology data (Table 1) to the catalytic and reverse catalytic models and selected the better-fit model according to AIC value. The catalytic model assumes lifelong seropositivity; the reverse catalytic model assumes a seropositive response that wanes over time. Serologic data from cattle and Père David’s deer fit the reverse catalytic models better (Figs 7 and 8). The duration of natural seropositivity was estimated to be 2 years for cattle and 1 year for Père David’s deer using TP data, and 4 years for Père David’s deer using AP data (Table 2). Serologic data from white-tailed deer fit the catalytic model better, which indicates lifelong seroconversion after infection (Fig 9). While the same model was selected regardless of whether the AP or TP data were fitted, the estimates for the parameters differed (Table 2). These results indicate that using AP data may identify the appropriate biological mechanism for which the parasite persists in the population, but control strategies based on AP may be biased due to the underestimation or overestimation of the parameters. Therefore, seropositivity wanes for cattle and Père David’s deer–with the duration of seropositivity varying by species–while seropositivity lasts for the entire lifespan in white-tailed deer.

**Table 2:** Parameter estimates from best-fit model with apparent (AP) versus true prevalence (TP).

*Species*	*Data*	*Model selected*	*Force of infection* *(years^-1^)*	*Duration of natural sero-positivity (years)*	*Maternal sero-positivity duration (years)*	*Reproduction number*	*Critical proportion to vaccinate*
***Cattle***	AP	Reverse Catalytic	0.051	2	n/a	0.61	n/a
	TP	Reverse Catalytic	0.055	2	n/a	0.66	n/a
***Pere David’s deer***	AP	Reverse Catalytic ^a^Mat	0.55	4	1.5	8.85	0.89
	TP	Reverse Catalytic ^a^Mat	0.41	1	1.5	6.53	0.85
***White-tailed deer***	AP	Catalytic ^a^Mat	0.135	lifelong	1.5	0.88	n/a
	TP	Catalytic ^a^Mat	0.170	lifelong	1.5	1.11	0.09

^a^Mat is defined as the antibodies found in less than 2 year old individuals. Those antibodies could be maternal antibodies or fetal antibodies from congenital infection with *N*. *caninum*.

To quantify the duration of maternal antibody persistence for both deer species, we fit reverse catalytic models with maternal immunity to the Père David’s deer serology data and catalytic models with maternal immunity to the white-tailed deer data to see if they fit the serology data better than their corresponding models without maternal immunity. The models with maternal immunity fit the data better and were selected, by AIC score, over the models did not assume maternal antibody presence (Tables E and F in S1 Text). The duration of maternal antibody persistence was estimated at 1.5 years for both deer species, regardless of whether AP or TP data were used (Table 2).

To determine if transmission dynamics are age-structured in all three species, we fit models with b-splined FoI and constant FoI to serologic data for each host species. The models without age-structured transmission were selected for all populations, indicating that the FoI was constant for all ages in all species (Table 2). This indicates, that the main route of transmission, likely, is vertical with a possibility of low rates of horizontal transmission.

To quantify the constant FoI in all host species, we fit both AP and TP serologic data to the best fit model selected by AIC. We found that Père David’s deer had the highest FoI, between 0.41 yr^-1^ and 0.55 yr^-1^. Cattle demonstrated the lowest FoI, between 0.051 yr^-1^ and 0.055 yr^-1^, and white-tailed deer had intermediate FoI estimates, between 0.135 yr^-1^ and 0.170 yr^-1^. There are clear differences in FoI estimates for each species.

To determine if each species could sustain chains of transmission only within their species, we calculated R_t_. When this value is greater than one, we assume that the species can maintain chains of transmission internally; when this value is less than one, we assume that the species cannot maintain chains of transmission internally. We found that Père David’s deer R_t_ estimates were 7 to 9, while cattle R_t_ estimates were <1. White-tailed deer R_t_ estimates were ~1; however, this species had an estimate of R_t_ >1 when TP was used. Based on these estimates, the two deer species seem to maintain chains of transmission within their populations, while cattle do not appear to be capable of maintaining transmission without an outside source (Table 2).

## Discussion

Our goal was to describe host species heterogeneity in the epidemiology of a multi-host pathogen, *N*. *caninum*, circulating in a community. As in many natural settings, our study area supports diverse livestock and wildlife species. This community structure is rarely investigated along an epidemiological continuum with the idea that each species might affect pathogen transmission. These kinds of studies are difficult to achieve, since studies designed to obtain detailed data measuring longitudinal transmission heterogeneities in the field could be prohibitively cost- and time-intensive. However, we have demonstrated that the most common and easily gathered data, serology and age, can be used to test hypotheses regarding transmission heterogeneities using mathematical models. Our results show that immunity in cattle and Père David’s deer wanes over time; for white-tailed deer, immunity appears to be lifelong. For white-tailed deer, natural boosting of the immune system might be occurring or natural infections may truly result in lifelong immunity to re-infection.

Our molecular data on white-tailed deer corroborates the model findings: none of the white-tailed deer tissue that was molecularly tested was positive; thus, seropositive individuals were either exposed rather than infected at sampling or detection was limited due to a low load of parasites and the arbitrary selection of tissue tested rather than focused in histopathology results. Another reason for the discrepancy of molecular and serological results of white-tailed deer was the possibility of cross-reaction with antibodies. However, the cELISA test uses the 65-kDa *N*. *caninum* tachyzoite antigen and a monoclonal antibody (MAb 4A 4–2) to detect antibodies in sera instead of whole tachyzoite antigens used in indirect ELISA and IFA tests, which largely alleviates the problem of cross-reaction with antibodies produced by antigenically related parasites such as *Toxoplasma gondii*, *Sarcocystis cruzi*, *Sarcocystis hominis*, and *Sarcocystis hirsute [30, 40].*

The reproductive number, R_t_, for cattle was below the threshold for sustaining internal epidemic transmission, suggesting that infections are the result of exposure to an outside source. For cattle, models with maternal antibodies were eliminated because sampled individuals were at least two years of age, and therefore beyond the lifespan of maternal antibodies. Unlike cattle, Père David’s deer and white-tailed deer could both maintain continuous chains of transmission (R*t* > 1) within their populations, presumably via vertical transmission. For Père David’s deer and white-tailed deer the models with maternal antibodies fitted better the data. These results may reflect the extent of colostrum consumption or in utero exposure with fetal antibody production. Our results indicate that transmission and immunodynamics of this pathogen are complex and differ by host species.

Differences in *N*. *caninum* seroprevalence, rates of abortion and immune responses between cattle breeds have been suggested [15, 16, 41, 42]. A proportion of each of the three ruminant populations sampled were in various stages of gestation. However, neither breed, reproductive status, nor gestational age was associated with the probability of being seropositive. We were unable to test associations for Père David’s deer; however, a previous retrospective study on *N*. *caninum* seroprevalence in the Père David’s deer herd found no correlation between calving rates and seropositivity [43].

Previously, French et al., 1999 studied transmission and control options of *N*. *caninum* in cattle. One of their remarkable findings was that, even with a high vertical transmission probability, some horizontal transmission was needed to maintain the endemism of the parasite in a cattle herd [44], a finding that is supported by our results (R_t_ <1 in cattle). Furthermore, seroprevalence studies in cattle, buffalo and Père David’s’ deer have suggested both age-specific and constant *N*. *caninum* prevalence with age [43, 45–48]. In our study none of the three ruminant populations showed age-structured FoI. This suggests that transmission within each of these populations is mainly vertical with low rates of horizontal transmission. High rates of horizontal transmission would be suspected if prevalence had increased with age or increased and decreased due to waning immunity and re-infection. Moreover, previous work in our study area found an estimated low environmental prevalence of *N*. *caninum* oocysts shed by wild canid hosts, which supports the hypothesis that transmission from canids to ruminants is infrequent [23].

Ecologically informed targeted control has been suggested for other multi-host pathogen systems: for example, targeting canines to control rabies in the Serengeti ecosystem [7] and targeting asymptomatically infected sheep to control peste des petits ruminants in multi-host communities [8]. Because results from our study suggest that vertical transmission is the main route by which *N*. *caninum* persists in the community, targeted control should be directed at the species of interest. For example, cattle would be the population of interest for farmers, while Père David’s deer would be the population of interest for conservationists. Specifically, our results suggest that, for cattle, controlling outside sources of infection (purchased animals and canid feces) may be helpful in controlling disease. However, control of outside sources would not likely affect disease transmission in either deer species, because these species can maintain chains of transmission within their populations. We also showed that immunity in cattle and Père David’s deer wanes over time, suggesting that boosting immunity with vaccines might prevent re-infection within these populations.

Currently, there is no vaccination for *N*. *caninum* and the development of a vaccine is not trivial. *N*. *caninum* is an obligatory intracellular parasite that seems to avoid host immunity by differentiating from tachyzoites into bradyzoites within cysts (tissue cysts). That differentiation changes the health status of a host from being acutely to chronically infected, and chronically infected individuals appear to be asymptomatic. However, during pregnancy, recrudescence of bradyzoites back to tachyzoites is plausible, resulting in symptomatic individuals. Pregnancy is associated with changes in the immune system, which can allow the parasite to infect the fetus. Therefore, candidate vaccines should prevent tachyzoite proliferation and dissemination to avoid transplacental transmission, and prevent tissue cyst formation [49]. Although various vaccine strategies (live attenuated vaccines, killed parasite lysates, total antigens or antigen fractions from killed parasites, and subunit vaccines) have been investigated to mediate protection in mice and cattle models, there are still disadvantages such as cost of production, safety, and stability that limit their application [20]. However, as one of the most economically important pathogens for the cattle industry with a global distribution, vaccine development for *N*. *caninum* should still be a priority.

Our study had a number of limitations. For instance, differences in the magnitude of the FoI per species captured species-specific differences in disease transmission. Despite that, the FoI cannot distinguish individual mechanisms that affect the health status of the individual. In addition, these models relate to the transition from sero-negative to sero-positive and does not assume that sero-positive animals are infectious. Therefore, conclusions about the mechanism behind the transmission cannot be done. To better confirm our findings a further evaluation incorporating a larger sample size for white-tailed deer and cattle is suggested. It would be interesting to study the context of optimal control strategies, and determine if they change regionally. Nevertheless, our conclusions hold for our studied populations, the mathematical models have previously illustrated their functionality with this type of data, and our results agree with previously-published, peer-reviewed literature.

Differentiating reservoirs from maintenance populations along the epidemiological continuum is an important step towards disease control and prevention in both domestic and wildlife populations. The methods we have demonstrated suggest which populations maintain disease (deer) and which require outside introduction of the pathogen (cattle). To our knowledge, this is the first study to suggest control and prevention strategies based on a better understanding of the heterogeneity of transmission across species. These methods guide targeted control and prevention by suggesting in which species biosecurity (cattle) or vaccination (cattle and Père David’s deer) would be helpful, with implications for other multi-host pathogens as well.

## Supporting information

S1 Text **S1 A Table. Sample size calculation of cattle.** The sample size calculation was done by Epi Info 7. The beef livestock population estimate comes from the sum of the number of farms at the township level multiplied by 50 (the estimated number of individuals per farm). Those farm estimates were given by OSU extension personnel at Muskingum, Morgan, Noble and Guernsey counties. We are using 24% of prevalence reported at ODA/ADDL with a 6% of precision (18–30%), 95% Confidence intervals. Therefore, we are requesting to sample up to 199 cattle at the Auction. The number of individuals to sample by township at the auction was calculated by weighting the total sample by the livestock population size at the township level. **S1 B Table. Stratified sample size of cattle by township. S1 C Table. Breeds of cattle sampled. S1 D Table. Cattle AIC values of all 16 models with apparent and true prevalence data. S1 E Table. Père David’s deer AIC values of all 16 models with apparent and true prevalence data. S1 F Table. White-tailed deer AIC values of all 16 models with apparent and true prevalence data.**
(DOCX)
