# Supplementary material for: Host species heterogeneity in the epidemiology of Nesopora caninum
Source: PLoS One. 2017 Aug 29;12(8):e0183900. doi: 10.1371/journal.pone.0183900 (PMC5574622; doi:10.1371/journal.pone.0183900)
Supplement: S1 Text — S1 A Table. Sample size calculation of cattle. The sample size calculation was done by Epi Info 7. The beef livestock population estimate comes from the sum of the number of farms at the township level multiplied by 50 (the estimated number of individuals per farm). Those farm estimates were given by OSU extension personnel at Muskingum, Morgan, Noble and Guernsey counties. We are using 24% of prevalence reported at ODA/ADDL with a 6% of precision (18–30%), 95% Confidence intervals. Therefore, we are requesting to sample up to 199 cattle at the Auction. The number of individuals to sample by township at the auction was calculated by weighting the total sample by the livestock population size at the township level. S1 B Table. Stratified sample size of cattle by township. S1 C Table. Breeds of cattle sampled. S1 D Table. Cattle AIC values of all 16 models with apparent and true prevalence data. S1 E Table. Père David’s deer AIC values of all 16 models with apparent and true prevalence data. S1 F Table. White-tailed deer AIC values of all 16 models with apparent and true prevalence data. (DOCX) [file pone.0183900.s001.docx]

Supplementary File for

Host species heterogeneity in the epidemiology of *Nesopora caninum*

Karla I. Moreno-Torres, Laura W. Pomeroy, Mark Moritz, William Saville, Barbara Wolfe, Rebecca Garabed

S1A Table. Sample size calculation of cattle.

| Total Population (beef livestock) | Expected frequency | Confidence limits % | Confidence level % | Total sample |
| --- | --- | --- | --- | --- |
| 19150 | 24 | 6 | 95 | 199 |

The sample size calculation was done by Epi Info 7. The beef livestock population estimate comes from the sum of the number of farms at the township level multiplied by 50 (the estimated number of individuals per farm). Those farm estimates were given by OSU extension personnel at Muskingum, Morgan, Noble and Guernsey counties. We are using 24% of prevalence reported at ODA/ADDL with a 6% of precision (18-30%), 95% Confidence intervals. Therefore we are requesting to sample up to 199 cattle at the Auction. The number of individuals to sample by township at the auction was calculated by weighting the total sample by the livestock population size at the township level.

S1B Table. Stratified sample size of cattle by township..

| **Number** | **County** | **Township** | **No. farms** | **No. cattle** | **Sample Size** | **Sampled** |
| --- | --- | --- | --- | --- | --- | --- |
| 1 | Guernsey | Jackson | 10 | 500 | 5 | 5 |
| 2 | Guernsey | Richland | 12 | 600 | 6 | 4 |
| 3 | Guernsey | Spencer | 15 | 750 | 8 | 9 |
| 4 | Guernsey | Valley | 11 | 550 | 6 | 0 |
| 5 | Guernsey | Westland | 12 | 600 | 6 | 6 |
| 6 | Morgan | Bloom | 8 | 400 | 4 | 4 |
| 7 | Morgan | Bristol | 2 | 100 | 1 | 0 |
| 8 | Morgan | Center | 12 | 600 | 6 | 0 |
| 9 | Morgan | Malta | 1 | 50 | 1 | 6 |
| 10 | Morgan | Manchester | 2 | 100 | 1 | 1 |
| 11 | Morgan | Meigsville | 3 | 150 | 2 | 4 |
| 12 | Morgan | Morgan | 10 | 500 | 5 | 2 |
| 13 | Morgan | York | 10 | 500 | 5 | 2 |
| 14 | Muskingum | Blue Rock | 25 | 1250 | 13 | 12 |
| 15 | Muskingum | Harrison | 35 | 1750 | 18 | 8 |
| 16 | Muskingum | Meigs | 1 | 50 | 1 | 1 |
| 17 | Muskingum | Rich Hill | 1 | 50 | 1 | 2 |
| 18 | Muskingum | Salt Creek | 35 | 1750 | 18 | 17 |
| 19 | Muskingum | Perry | 20 | 1000 | 10 | 2 |
| 20 | Muskingum | Union | 40 | 2000 | 20 | 20 |
| 21 | Muskingum | Wayne | 20 | 1000 | 10 | 7 |
| 22 | Noble | Brooksfield | 1 | 50 | 1 | 0 |
| 23 | Noble | Buffalo | 6 | 300 | 3 | 0 |
| 24 | Noble | Center | 23 | 1150 | 12 | 10 |
| 25 | Noble | Noble | 9 | 450 | 5 | 6 |
| 26 | Noble | Olive | 54 | 2700 | 27 | 5 |
| 27 | Noble | Seneca | 3 | 150 | 2 | 2 |
| 28 | Noble | Sharon | 1 | 50 | 1 | 1 |
| 29 | Noble | Wayne | 1 | 50 | 1 | 1 |
| TOTAL |  |  | 383 | 19150 | 199 | 137 |

S1C Table. Breeds of cattle sampled.

| **Breed** | **Number sampled** |
| --- | --- |
| **Beef** | **89** |
| Black Angus | 50 |
| Red Angus | 6 |
| Charolais | 3 |
| Hereford | 11 |
| Shorthorn | 7 |
| Simmental | 12 |
| **Dairy** | **14** |
| Brown Swiss | 1 |
| Guernsey | 2 |
| Holstein | 6 |
| Jersey | 5 |
| **Crossbreed** | **34** |
| Angus and Charolais | 3 |
| Angus and Hereford | 5 |
| Angus and Simmental | 5 |
| Angus and Holstein | 5 |
| Angus cross | 7 |
| Charolais cross | 1 |
| Hereford cross | 3 |
| Maine cross | 1 |
| Shorthorn cross | 1 |
| Simmental cross | 2 |
| Simmental and Charolais | 1 |

S1D Table. Cattle AIC values of all 16 models with apparent and true prevalence data.

| Species |  | Data | Model | AIC |
| --- | --- | --- | --- | --- |
| Cattle |  | AP | Catalytic | 31.0162 |
|  |  | AP | Reverse Catalytic | 29.5784 |
|  |  | AP | Catalytic Mat | 33.3462 |
|  |  | AP | Reverse Catalytic Mat | 31.5784 |
|  |  | AP | Catalytic Age | 38.2939 |
|  |  | AP | Reverse Catalytic Age | 34.8141 |
|  |  | AP | Catalytic Mat Age | 35.6257 |
|  |  | AP | Reverse Catalytic Mat Age | 45.3528 |
|  |  | TP | Catalytic | 29.7305 |
|  |  | TP | Reverse Catalytic | 28.1471 |
|  |  | TP | Catalytic Mat | 32.0387 |
|  |  | TP | Reverse Catalytic Mat | 30.1471 |
|  |  | TP | Catalytic Age | 37.7526 |
|  |  | TP | Reverse Catalytic Age | 36.8145 |
|  |  | TP | Catalytic Mat Age | 31.6456 |
|  |  | TP | Reverse Catalytic Mat Age | 40.0188 |

S1E Table. Père David’s deer AIC values of all 16 models with apparent and true prevalence data.

| Species | Data | Model | AIC |
| --- | --- | --- | --- |
| Père David’s deer | AP | Catalytic | 40.8318 |
|  | AP | Reverse Catalytic | 32.0546 |
|  | AP | Catalytic Mat | 35.8637 |
|  | AP | Reverse Catalytic Mat | 31.2982 |
|  | AP | Catalytic Age | 39.1229 |
|  | AP | Reverse Catalytic Age | 40.9799 |
|  | AP | Catalytic Mat Age | 35.7538 |
|  | AP | Reverse Catalytic Mat Age | 64.07 |
|  | TP | Catalytic | 27.5434 |
|  | TP | Reverse Catalytic | 23.3347 |
|  | TP | Catalytic Mat | 25.0684 |
|  | TP | Reverse Catalytic Mat | 23.2363 |
|  | TP | Catalytic Age | 29.2525 |
|  | TP | Reverse Catalytic Age | 30.2108 |
|  | TP | Catalytic Mat Age | 29.2279 |
|  | TP | Reverse Catalytic Mat Age | 31.4016 |

S1F Table. White-tailed deer AIC values of all 16 models with apparent and true prevalence data.

| Species | Data | Model | AIC |
| --- | --- | --- | --- |
| White-tailed deer | AP | Catalytic | 30.8464 |
|  | AP | Reverse Catalytic | 25.7357 |
|  | AP | Catalytic Mat | 17.5112 |
|  | AP | Reverse Catalytic Mat | 19.7417 |
|  | AP | Catalytic Age | 33.167 |
|  | AP | Reverse Catalytic Age | 30.809 |
|  | AP | Catalytic Mat Age | 25.9275 |
|  | AP | Reverse Catalytic Mat Age | 24.8298 |
|  | TP | Catalytic | 24.63 |
|  | TP | Reverse Catalytic | 21.6594 |
|  | TP | Catalytic Mat | 16.0035 |
|  | TP | Reverse Catalytic Mat | 17.8669 |
|  | TP | Catalytic Age | 28.9082 |
|  | TP | Reverse Catalytic Age | 28.0131 |
|  | TP | Catalytic Mat Age | 22.0322 |
|  | TP | Reverse Catalytic Mat Age | 23.9069 |
